# Social skills training using multiple humanoid robots for individuals with autism spectrum conditions

**DOI:** 10.3389/fpsyt.2023.1168837

**Published:** 2023-07-19

**Authors:** Keiji Takata, Yuichiro Yoshikawa, Taro Muramatsu, Yoshio Matsumoto, Hiroshi Ishiguro, Masaru Mimura, Hirokazu Kumazaki

**Affiliations:** ^1^Department of Psychology, Saitama Gakuen University, Saitama, Japan; ^2^Department of Systems Innovation, Graduate School of Engineering Science, Osaka University, Osaka, Japan; ^3^Department of Neuropsychiatry, Keio University School of Medicine, Tokyo, Japan; ^4^Human Augmentation Research Center, National Institute of Advanced Industrial Science and Technology, Chiba, Japan; ^5^Department of Clinical Research on Social Recognition and Memory, Research Center for Child Mental Development, Kanazawa University, Ishikawa, Japan; ^6^College of Science and Engineering, Kanazawa University, Ishikawa, Japan; ^7^Department of Neuropsychiatry, Graduate School of Biomedical Sciences, Nagasaki University, Nagasaki, Japan

**Keywords:** social skills training, autism spectrum conditions, perspective of others, empathy skills, humanoid robot

## Abstract

**Introduction:**

Social skills training (SST) is used to help individuals with autism spectrum conditions (ASC) better understand the perspectives of others and social interactions, develop empathy skills, and learn how to engage with others socially. However, many individuals with ASC cannot easily sustain high motivation and concentration during such an intervention when it is administered by humans. We developed a social skills training program using multiple humanoid robots (STUH), including an android robot, that aimed to enable individuals with ASC to become familiar with the perspectives of others and improve their sociability and empathy skills. The objective of the present study was to investigate the effectiveness of STUH for these individuals.

**Methods:**

In STUH, we prepared 50 social exercises that consisted of conversations and behavioral interactions between an android robot and a simple humanoid robot. We prepared another humanoid robot that featured a cartoon-like and mechanical design, which played the role of host. In the first half-session of STUH, participants worked on the exercise from the perspective of an outsider. In the second half-session of STUH, they simulated experience by using robots as their avatars. The intervention associated with STUH was conducted for five days in total. We conducted an analysis of variance (ANOVA) featuring the intervention time point as the independent variable to examine changes in each score on the sociability index items.

**Results:**

In total, 14 individuals with ASC participated in the study. The results of multiple comparison tests using the Bonferroni method indicated that all sociability index items improved between preintervention and follow-up. Our program enabled the participants to become familiar with the perspectives of others and improve their sociability.

**Discussion:**

Given the promising results of this study, future studies featuring long-term follow-up should be conducted to draw definitive conclusions about the efficacy of our training system.

## 1. Introduction

Autism spectrum conditions (ASC) is characterized by deficits in social communication and limited and repetitive behavioral patterns (1). Individuals with ASC experience profound difficulties in the social domain, such as difficulties with interpersonal skills. Social difficulties lead to poor functional outcomes among these individuals even when there is no coexisting intellectual disability: increased risks of bullying (2), decreased access to employment, independent living, longstanding friendships, and intimate relationships (3), and a lower quality of life in general (4). Social skills training (SST) is an intervention based on social learning theory that encompasses a series of behavioral strategies aimed at teaching new skills to overcome social difficulties (5). This method is used to help individuals with ASC better understand the perspectives of others and social interactions, develop empathy skills and learn to engage with others socially. With regard to the success of SST, repetitive learning and learning from other perspectives are the most important factors.

The use of SST for individuals with ASC has been received a great deal of attention (6, 7). However, these results should be interpreted cautiously due to the moderate quality of the available evidence (6). Individuals with ASC often cannot attribute mental states to themselves and others [theory of mind (ToM)] (8). Consequently, they are unaware of how their behavior affects others (9), which is connected to their lack of enthusiasm for trainings conducted by humans (10). A study reported that robotic intervention promoted joint attention in individuals with ASC better than did interventions conducted by a human trainer (11). In addition, their intensive sensory processing may be affected by the human dynamic facial features and expressions, which are likely to trigger sensory and emotional overstimulation and distractions (12). This situation can interfere with their learning regarding other perspectives and the repeated training associated with SST, as they tend to avoid sensory stimulations actively and focus instead on more predictable, rudimentary features. A more effective intervention with a greater substantive impact on social skills is urgently needed.

Increasing anecdotal evidence attests to the fact that individuals with ASC may have special opportunities to use robots for such help (13–19). Robots can allow them to control and replicate a scene featuring smooth and exact conversation despite their reactions, thus contributing to a more structured and standardized intervention. Unlike human beings, humanoid robots, which operate within predictable and legal systems, provide a highly structured study environment to them, thus encouraging them to focus on relevant stimulus. Structured interactions with robots are high likelihood that lead to the emergence of standardized social conditions in which certain social behaviors can occur (20, 21). Such robots never become tired and can ensure the repetition of exactly the same situation across instances; thus, interventions using robots are suitable for facilitating repetitive learning on the part of individuals with ASC.

A previous study revealed that using multiple robots provides individuals with ASC flexible and realistic role-play scenarios, promoting concentration during the evaluation and encouraging high levels of motivation from start to finish (22). In another study, multiple robots allow participants to practice social skills in a safe setting, facilitate the repetition of tasks, and support generalizability (23). Roleplay exercises with robots can promote mental simulation pertaining to social events, thus potentially offering greater insight into minds. A previous study revealed that the use of multiple humanoid robots enables the participant to put himself in the position of each robot and to become familiar with the perspectives of others (24). Interventions using multiple robots could be effectively used to provide innovational SST to individuals with ASC.

One concern in this context is that the acquisition of skills through the help of robotic intervention does not allow those skills to be generalized to daily life. The appearance and behaviors of an android robot resemble those of an actual human (25, 26). Android robots exhibit a variety of facial expressions (e.g., smiling, nodding, and forehead movements) during conversation and can offer subtle non-verbal cues. Therefore, it is possible that the establishment of intelligent three-dimensional their study environments using android robots may represent a powerful means for enhancing skills that can be generalized to real-world settings (10). In fact, previous studies using android robots found that such skills were generalizable to daily life (25, 26).

In light of these factors, we developed a social skills training program using multiple humanoid robots (STUH), including an android robot, that aimed to help individuals with ASC become familiar with the perspectives of others and improve their sociability and empathy skills. In STUH, we prepared 50 social exercises that consisted of conversations and behavioral interactions between android robots and simple humanoid robots. Previous studies suggested that individuals with ASC have different preferences for the appearance of robots (27), with each type of robot possessing different advantages (16). Consequently, we opted to utilize three different types of robots in this study. We prepared another humanoid robot that featured a cartoon-like and mechanical designthis robot played the role of host. STUH includes four themes: emotion guessing in a one-on-one conversational situation, emotion guessing in a multiperson conversational situation, interpersonal manner, and empathic response to a person in distress. STUH is composed of a first half-session and a second half-session, each of which is composed of five exercises. In the first half-session, participants worked on the exercise from the perspective of an outsider. In the second half-session, they simulated experience by using robots as their avatars. Our system establishes a structured environment in which participants can learn and practice their social skills. The purpose of the present study was to investigate the effectiveness of STUH in individuals with ASC.

## 2. Materials and methods

### 2.1. Participants

The present study was conducted with the approval of the Ethics Committee of Kanazawa University. Participants were recruited through leaflets explaining the details of the experiment. All procedures performed in studies involving human participants were conducted in accordance with the ethical standards of the institutional and/or national research committee and the 1964 Declaration of Helsinki and its subsequent amendments or equivalent. After receiving a full explanation of the study, all participants and their guardians agreed to participate in the study. Written informed consent for the release of any potentially personally identifiable images or data contained in this article has been obtained from the individual and/or the legal guardian of the minor. No conflicts of beha exist in this study. The inclusion criteria included (1) having a diagnosis of autism spectrum disorders (ASD) according to the Diagnostic and Statistical Manual of Mental Disorders, Fifth Edition (DSM-5;1) from the experienced psychiatrist, (2) having an IQ ≥ 70, and (3) not on any medication. The exclusion criteria were medical conditions associated with ASD (e.g., Shank3, fragile × syndrome, and Rett syndrome). To exclude other psychiatric diagnoses, the Mini-International Neuropsychiatric Interview (28) was performed. At the time of registration, the diagnoses of all participants were confirmed by an experienced psychiatrist with more than 15 years in ASD using the criterion contained in the DSM-5 and standardized criteria drawn from the Diagnostic Interview for Social and Communication Disorders (DISCO) (29). The DISCO has been reported that they have good psychometric properties (30). All participants had been acquainted with each other for more than 1 year.

Participants did the Autism Spectrum Quotient-Japanese version (AQ-J) (31), which has been used to assess behaviors and traits specific to ASD. The AQ-J is a brief questionnaire including five subscales (social skills, attention switching, attention to detail, imagination, and communication). Prior work with the AQ-J has been depicted across cultures (32) and ages (33, 34). Notably, the AQ is sensitive to the broader autism phenotype. In this study, we did not set a cutoff based by AQ-J score and used only the DSM-5 and DICSO to diagnose ASD and to determine whether or not to include participants in our study.

Full-scale IQ scores were measured by either using the Wechsler Adult Intelligence Scale–Third Edition (WAIS-III) or the Japanese Adult Reading Test (JART) (35). We used both the WAIS-III and JART in this study because they have comparable results. The latter is a standardized cognitive function test used to estimate the premorbid IQ of individuals with cognitive impairments. The JART has validity with respect to measuring IQ. The JART results can be compared to those of the WAIS-III (35).

The severity of participant’s social anxiety traits was measured by using the Liebowitz Social Anxiety Scale (LSAS) (36). This clinician-led scale makes up of 24 items, including 13 items describing performance situations and 11 items describing social interaction situations. Each item was rated separately for “fear” and “avoidance” on a 4-point categorical scale. Receiver operating curve (ROC) analyses showed that an LSAS score of 30 is correlated with minimal traits and is the optimal cutoff value for distinguishing between individuals with and without social anxiety disorder (37).

The Adolescent/Adult Sensory Profile [AASP; (38)] is a self-administered questionnaire used to measure sensory processing in individuals aged 11 years and up. The internal consistency coefficients for the AASP range from 0.64 to 0.78 on the quadrant scores. Participants indicated how often they exhibited certain behaviors in relation to sensory experiences on a scale of one (“almost never”) to five (“almost always”). The AASP examines four different “quadrants” of sensory processing: low registration, sensation seeking, sensory sensitivity, and sensation avoiding. Because the AASP does not classify responses according to individual “perceptual domains” (such as the auditory, visual, or tactile domains), a perceptual domain analysis was not done for this study.

The Interpersonal Reactivity Index (IRI) is a multidimensional scale of empathic traits that includes 28 self-report items across four subscales [7 items each; (39)]. In this study, we used the Japanese version of the Interpersonal Reactivity Index (IRI-J), which has exhibited adequate reliability and construct validity (40). The current study uses all subscales. The personal distress scale focuses on the tendency to experience distress and discomfort in response to extreme distress in others (e.g., “In emergency situations, I feel apprehensive and ill-at-ease”). The empathic concern scale assesses the tendency to experience feelings of sympathy and compassion for others facing unfortunate situations (e.g., “I often have tender, concerned feelings for people who are less fortunate than me”). The perspective-taking scale measures the reported tendency to adopt the psychological perspectives of others spontaneously in everyday life (e.g., “I try to consider everybody’s side of a disagreement before I make a decision”). The fantasy scale measures the tendency to transpose oneself imaginatively into fictional situations (e.g., “I really get involved with the feelings of the characters in a novel”). Each item is answered on a 5-point Likert-type scale with responses ranging from 1 = strongly disagree to 5 = strongly agree.

In total, 14 individuals with ASC participated. The details are shown in Table 1. All participants completed the trial procedures without the emergence of any technological problems or participant distress, which would have led to session termination. Our research assistants observed the posture and reaction of the participants and measured them as performance in the experiment using a Likert scale. The ratings for posture and reaction of all participants were good and confirmed that all participants remained focused during the experiment and were highly motivated from the start to the end of it. Additionally, no signs of “survey fatigue” were observed among the participants in this study.

**TABLE 1:** Descriptive statistics of participants.

Characteristics	*n* = 14 M (SD)
Age (years)	17.57 (3.39)
Gender (Male: Female)	11:3
Full scale IQ	89.50 (10.95)
AQ-J	26.00 (7.06)
LSAS-J	51.93 (23.09)
**AASP**	
Low registration	39.07 (12.45)
Sensation seeking	35.50 (7.63)
Sensory sensitivity	38.14 (9.14)
Sensation avoiding	35.07 (6.39)

M, mean; SD, standard deviation. The only demographic factor applied as an inclusion criterion in this study was full-scale IQ.

### 2.2. Robotic system

**FIGURE 1:**
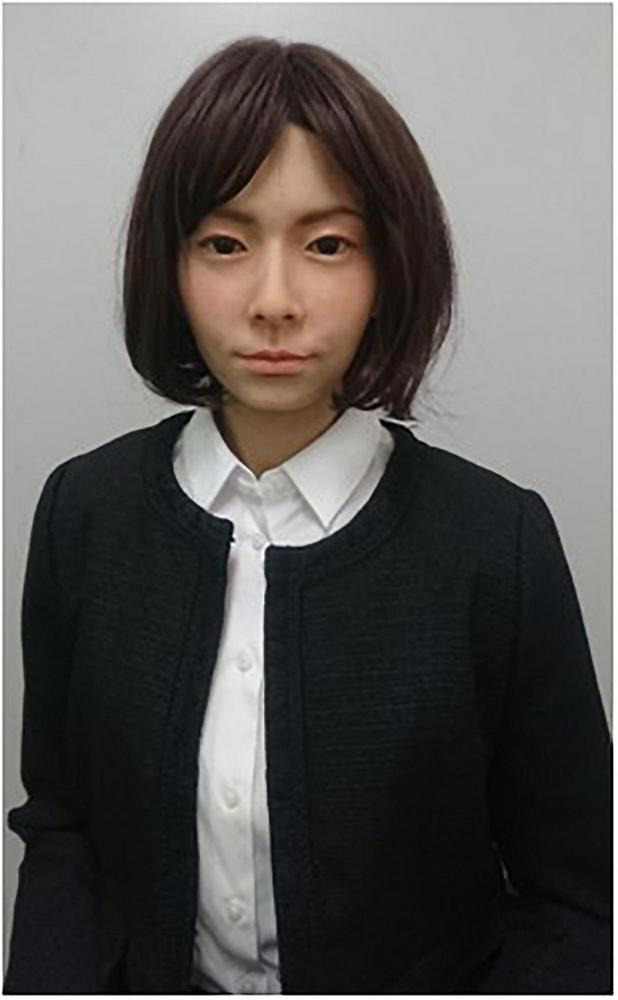
A-Lab android ST.

We used A-Lab android ST (Figure 1) (41, 42) (A-Lab Co., Ltd., Chiyoda-ku, Tokyo, Japan), which is a female android robot with an appearance similar to that of a real person. Its motor system consisted of air cylinders and rotary actuators with the 19 degrees of freedom (DoFs) to produce flexible and silent motions: 12 for facial expression such as smiling and brow movements, 4 for head motion such as nodding, and 3 for upper body motion such as breathing. Its utterance was created by synthesizing voice sound with a commercial Text-To-Speech (TTS) software and synchronously producing lip motion with it. It is teleoperated by a graphical user interface on a laptop computer including buttons to trigger producing a preregistered sequence of its motions and utterances. In this study, this robot was teleoperated by a research assistant in the first half-session. It was teleoperated by the participant in the second half-session. We chose the A-Lab android ST specifically for its ability to move its eyes, which is an important feature for role-playing interactions between robots. In addition, the use of android robots, which bear a resemblance to humans, offers the advantage of potentially generating more generalizable results.

**FIGURE 2:**
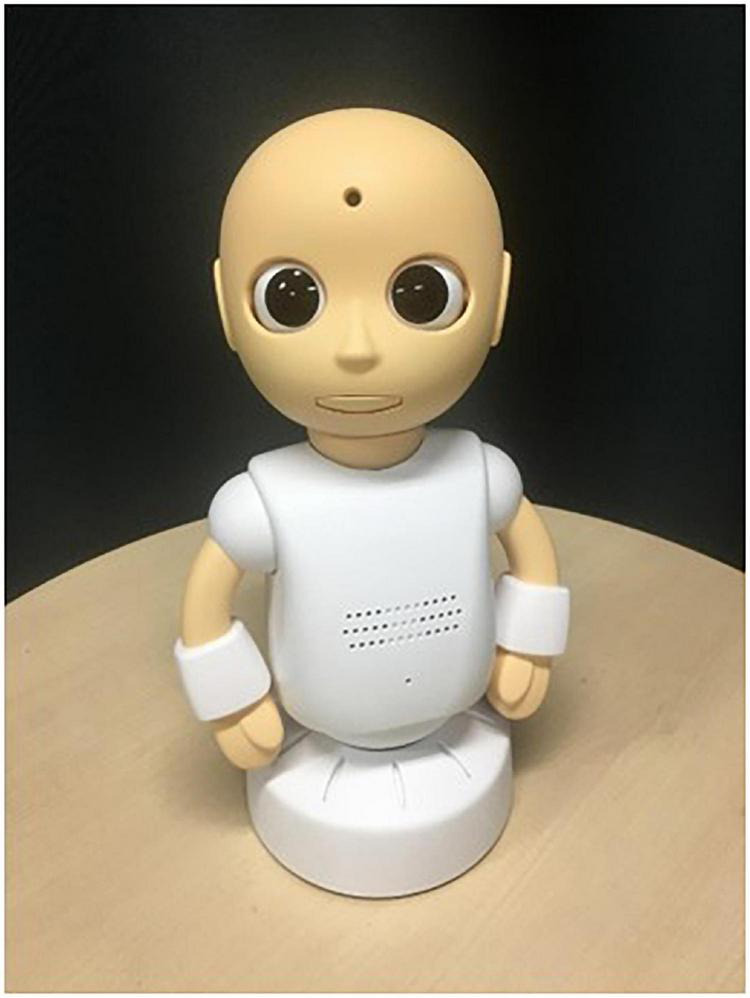
CommU.

A desktop-type, simple humanoid robot (CommU, Vstone Co., Ltd., Osaka city, Osaka, Japan) (Figure 2) (11, 43, 44) was used as another robotic performer that communicated with the above android robot in STUH. Its small size (304 mm) and childlike cute shape were expected to help preventing individuals with ASC to have fearfulness. Its motor system was driven with silent servo motors corresponding to the fourteen DoFs: waist (2), left shoulder (2), right shoulder (2), neck (3), eyes (3), eyelids (1), and lips (1). Note that its face was limited to produce a simplified expressions compared to real human face due to the lack of DoFs to move facial parts. Instead, the multiple DoFs were dedicated to produce rich expressions with its eye gaze. In this study, this robot was also teleoperated by a research assistant and the participant in the first and second half-session, respectively. The CommU robot was selected for its clear and articulate eye movements, which are essential for effective role-playing interactions between robots. In addition, the CommU robot can display various expressions despite its simplicity, making it highly suitable for SST on understanding emotional recognition.

**FIGURE 3:**
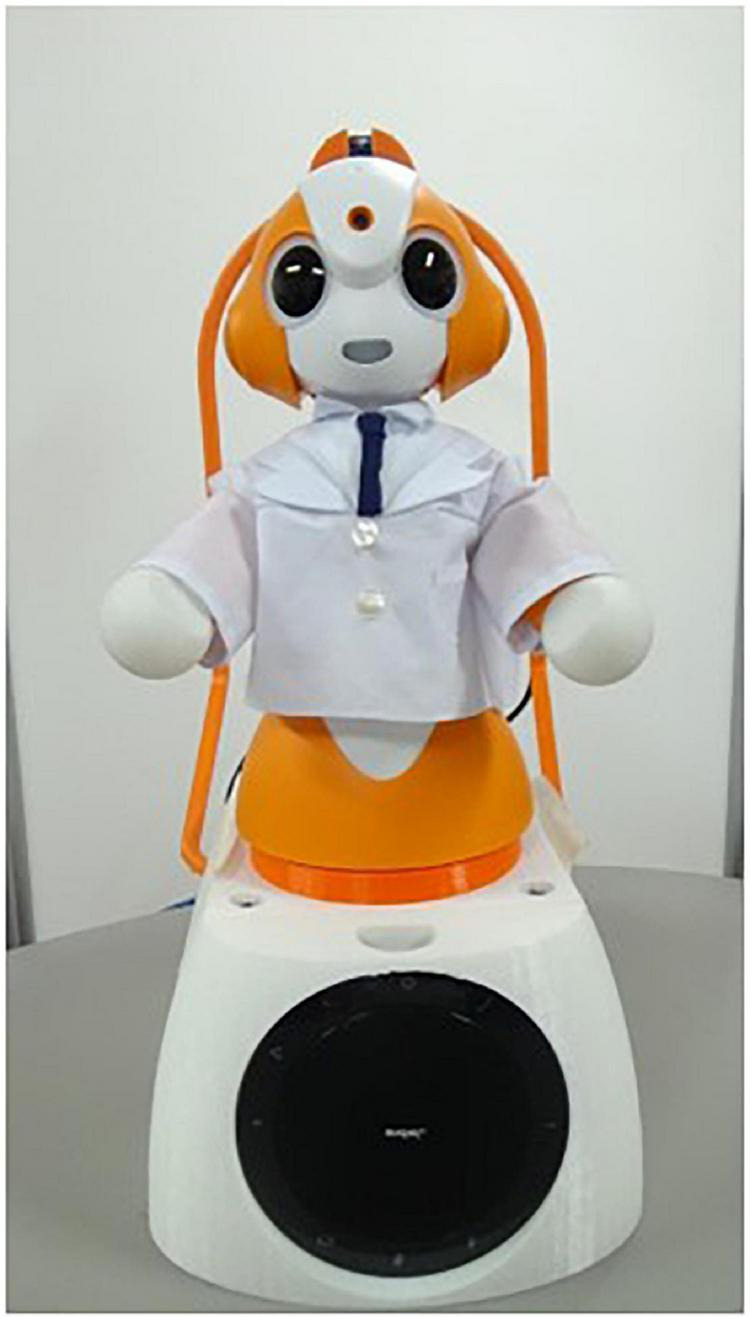
Sota.

Sota (Figure 3) (45–47) (Vstone Co., Ltd.) is another humanoid robot with a small (280 mm tall), cartoon-like and mechanical design, which is expected to help prevent fearfulness as in the case of CommU. Sota uses the same motor system as CommU but has fewer, namely, eight, DoFs: base (1), left shoulder (1) and elbow (1), right shoulder (1) and elbow (1), and neck (3). These DoFs enable this robot to exhibit non-verbal behavior such as nodding and watching. In the current experiment, Sota was teleoperated by a research assistant using a laptop computer to serve as a host for participants. Namely, through the computer interface, the experimenter can not only see and hear participants but also flexibly talk through Sota using a headset device and elicit its gaze and body gestures using a touch panel device. Note that some simple gestures are designed to be produced automatically in response to the experimenter’s words to convey a vivid presence as a speaker. Furthermore, the research assistant’s voice was captured by the laptop computer and converted to feature a high pitch that resembled the voice of a mechanical robot with the aim of mitigating the presence or influence of Sota’s human operator. In this study, this robot was teleoperated by a research assistant in both the first and second half-sessions. We selected the Sota robot due to its “cute” facial features and attractiveness, which could provide a pleasant atmosphere in its role as host.

### 2.3. Procedure

**FIGURE 4:**
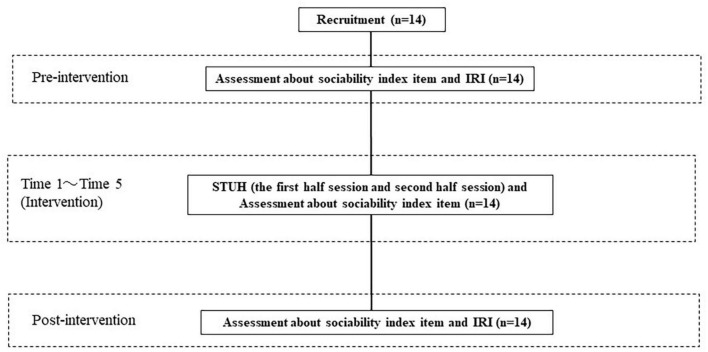
Flow chart. The intervention associated with STUH was conducted for 5 days. Preintervention (1 week before the experiment), after each intervention and at follow-up (2 weeks after the last session), participants rated the sociability index items. In addition, they were also rated on the IRI at preintervention and follow-up.

**FIGURE 5:**
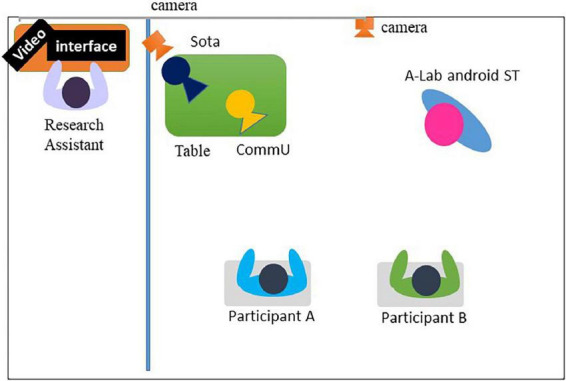
Experimental room setting during the first half-session. In the intervention booth, A-Lab android ST was placed in front of the participants to demonstrate the session. In addition, Sota played the role of host. These robots were teleoperated by research assistants. Two participants faced the robots.

**FIGURE 6:**
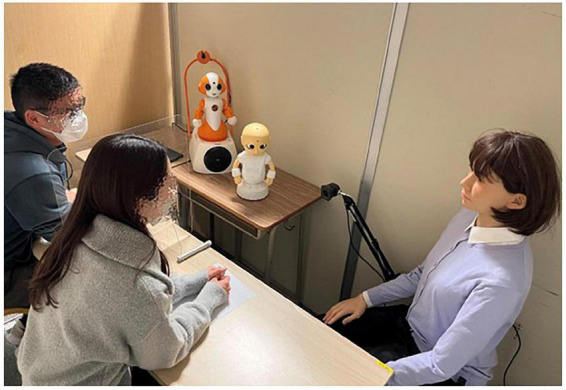
Image of the experimental setting during the first half-session.

The intervention associated with STUH each day (i.e., the first half-session and the second half-session) lasted approximately 1 h and was conducted over 5 days (i.e., Time 1, Time 2, Time 3, Time 4, and Time 5) in total. The average interval of each intervention was 2 weeks. Each day, participants received an intervention that was divided into the first half-session and the second half-session, each of which included of five exercises. The flow chart of this study is shown in Figure 4. In the first half-session of each exercise, Sota explained the content and purpose of the exercise and oriented the participants toward the content of the exercise. Sota also explained to the participants the social situation that the robots would demonstrate and the roles that each robot would play. Two participants experienced five exercises featuring interaction between A-Lab android ST and CommU, which were teleoperated by a research assistant who pressed the buttons in a predetermined order to ensure that the interaction between the robots took place at the same time. Participants worked in pairs and were instructed to focus on the robots’ movements, facial expressions, and conversations during the demonstration. In this study, participants were paired according to the compatibility of their hobbies. We also checked the participants’ opinions and experiences regarding the procedure. “On some occasions, pairs changed due to unforeseen circumstances (i.e., family misfortune, infection by COVID-19). Figures 5, 6 illustrate the experimental room setup in the first half-session. The persons in Figure 6 have given written and informed consent to publish this image. Example exercises are shown in Supplementary material 1. After each exercise, Sota asked participants to report the robot’s emotions and the causes of those emotions, to indicate what the appropriate attitudes and actions were in that social situation, and to identify the attitudes and actions that needed to be improved. After their report, Sota gave feedback for each participant’s report. One objective of the first half-session was to enable the participants to learn about the social situation in question and consider appropriate behavior from the perspective of an outsider.

**FIGURE 7:**
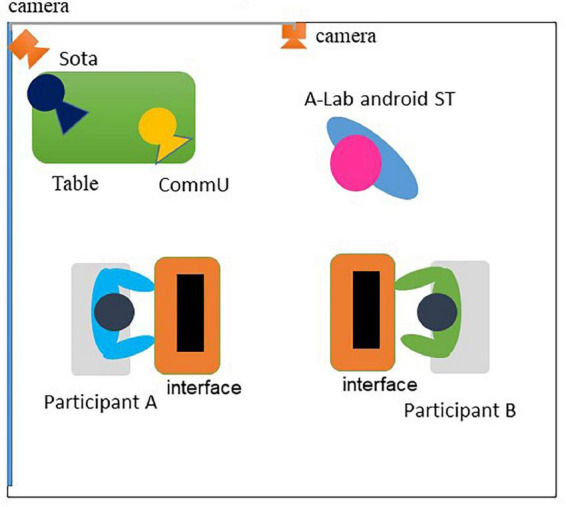
Experimental room setting during the second half-session. In the intervention booth, A-Lab android ST was placed in front of the participants to demonstrate the session. In addition, Sota played the role of host. These robots were teleoperated by participants. Each participant was located in front of a PC. Two participants faced each other.

After the first half-session, the participants were seated facing each other, and the computers used to operate the robots were placed in front of them. In this study, before the second half-session, the research assistant explained the operation of the robots (i.e., A-Lab android ST and CommU) and provided an example. After confirming that there were no questions regarding the operation of the robot, the second half-session of the intervention began. Figure 7 illustrates the experimental room setup in the second half-session. The participants pressed the buttons in a predetermined order to work through the exercises based on the interactions between A-Lab android ST and CommU. Example exercises are shown in Supplementary material 1 (i.e., the same script was used in the first half-session and the second half-session). One objective of the second half-session was to allow participants to experience a simulated social situation by teleoperating the robot and considering appropriate behavior from the perspective of the robot involved in the interaction.

Preintervention (1 week before the experiment), after each intervention and at follow-up (2 weeks after the last session), two raters independently rated the sociability index item pertaining to the participants’ understanding of and beliefs regarding the feelings, behaviors, and perceptions of others in daily life situations as noted below.

(1) Understand the other person’s way of thinking and situation.
(2) Understand the other person’s feelings.
(3) Understand the reason why the other person acted the way they did.
(4) Try to understand why they think the way they do when conversing with others who think differently.
(5) Even in cases of conflict, make an effort to put yourself in the other person’s shoes.
(6) When listening to others, consider what they want to say.
(7) Always try to put yourself in other people’s shoes to understand them.
(8) Do not criticize the other person without considering their perspective.

The score ranged from 1 (very poor) to 7 (very excellent). Prior to the experiment, both raters received training (lasting approximately 5 h) on scoring the interviews while watching videos of interview scenes. The score used in this study was the average of the scores of the two raters. The intraclass correlation coefficient was calculated to confirm interrater reliability. The coefficient was low but acceptable (ICC = 0.684). After the intervention, the following questions were asked of the participant’s supporters: “Did the participants learn to understand the perspective of the interviewer after the intervention?”

### 2.4. Statistical analysis

We conducted statistical analysis using SPSS version 24.0 software (IBM, Armonk, NY, USA). We conducted an analysis of variance (ANOVA) featuring the intervention time point as the independent variable to examine the changes in each score on the sociability index items.

The following analyses were conducted to examine the relationships between the intervention and the corresponding changes in empathy and autistic tendencies. First, a *t*-test was conducted on the IRI scores between preintervention and postintervention. Subsequently, the correlation coefficient between the change in IRI scores and the AQ-J was calculated. An alpha level of 0.05 was used for these analyses.

## 3. Results

Since this study had a small sample size, the Shapiro–wilk test for normality was conducted, and we confirmed that the data were parametric. Although normality was not confirmed for the sociability index items, all parametric tests were conducted in this study due to the fact that analysis of variance is robust against deviations from normality. A one-way ANOVA was conducted to examine the changes in each sociability index item score from preintervention to follow-up. The assumption of sphericity was not confirmed by all analyses, and so Huynh-Feldt’s correction was employed. Since the ANOVA was repeated for eight items, the Bonferroni correction was used to evaluate significance.

**FIGURE 8:**
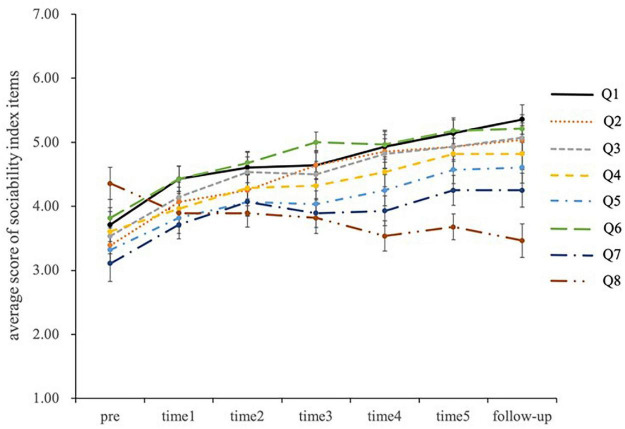
Changes in sociability index item scores with STUH.

The results indicated a significant main effect of measurement time in all sociability index items (*p* < 0.05). The results of multiple comparison tests using the Bonferroni method showed that all sociability index items improved from the preintervention assessment to follow-up evaluation. Descriptive statistics and the results of the ANOVA are presented in Table 2. The time series of changes in the scores of sociability index items associated with STUH is shown in Figure 8.

**TABLE 2:** Effects of the time of measurement on sociability index items.

	Time of Measurement							*F*	*p*	Mean difference (SE)	Multiple Comparisons using the Bonferroni Method
	Pre M (SD)	Time 1 M (SD)	Time 2 M (SD)	Time 3 M (SD)	Time 4 M (SD)	Time 5 M (SD)	Follow-up M (SD)				
Q1	3.71 (1.41)	4.43 (1.07)	4.61 (1.26)	4.64 (1.19)	4.93 (1.27)	5.14 (1.11)	5.36 (1.22)	13.72	<0.001	−1.64 (0.21)	Pre < Follow-up (*p* < 0.001, *d* = −1.34)
Q2	3.39 (1.42)	4.07 (1.22)	4.25 (1.27)	4.64 (1.13)	4.86 (1.38)	4.93 (1.05)	5.04 (1.20)	17.30	<0.001	−1.64 (0.20)	Pre < Follow-up (*p* < 0.001, *d* = −1.32)
Q3	3.54 (1.45)	4.14 (1.24)	4.54 (1.26)	4.50 (1.07)	4.82 (1.25)	4.93 (1.18)	5.07 (1.25)	12.42	<0.001	−1.54 (0.21)	Pre < Follow-up (*p* < 0.001, *d* = −1.23)
Q4	3.61 (1.62)	3.96 (1.23)	4.29 (1.18)	4.32 (1.16)	4.54 (1.23)	4.82 (1.28)	4.82 (1.31)	7.49	<0.001	−1.21 (0.23)	Pre < Follow-up (*p* < 0.001, *d* = −0.94)
Q5	3.32 (1.19)	3.82 (1.31)	4.07 (1.12)	4.04 (1.11)	4.25 (1.30)	4.57 (1.14)	4.61 (1.29)	10.46	<0.001	−1.29 (0.20)	Pre < Follow-up (*p* < 0.001, *d* = −1.06)
Q6	3.82 (1.52)	4.43 (1.03)	4.68 (0.95)	5.00 (0.86)	4.96 (1.20)	5.18 (1.06)	5.21 (1.13)	10.37	<0.001	−1.39 (0.22)	Pre < Follow-up (*p* < 0.001, *d* = −1.24)
Q7	3.11 (1.47)	3.71 (1.18)	4.07 (1.18)	3.89 (1.20)	3.93 (1.25)	4.25 (1.24)	4.25 (1.40)	6.98	<0.001	−1.14 (0.21)	Pre < Follow-up (*p* < 0.001, *d* = −0.89)
Q8	4.36 (1.34)	3.89 (1.13)	3.89 (1.17)	3.82 (1.28)	3.54 (1.26)	3.68 (1.06)	3.46 (1.37)	3.99	0.002	0.89 (0.21)	Pre > Follow-up (*p* < 0.001, *d* = 0.72)

M, mean; SD, standard division; SE, standard error. Q1. Understand the other person’s way of thinking and situation. Q2. Understand the other person’s feelings. Q3 Understand the reason why the other person acted the way they did. Q4. Try to understand why they think the way they do when conversing with others who think differently. Q5. Even in cases of conflict, make an effort to put yourself in the other person’s shoes. Q6. When listening to others, consider what they want to say. Q7. Always try to put yourself in other people’s shoes to understand them. Q8. Do not criticize the other person without considering their perspective.

To examine the efficacy of STUH with regard to improving empathy skills, a t test was conducted on the IRI subscore from preintervention to postintervention. The results showed no significant differences. The details are shown in Table 3. When the correlation coefficients between the change in each factor of the IRI subscore between preintervention and postintervention and the AQ-J scores were calculated, a positive correlation was found between the change in the fantasy scale subscore and the AQ-J scores (*r* = 0.56, *p* < 0.05). The results of the correlation analysis are shown in Table 4.

**TABLE 3:** Means and standard error of the mean IRI at preintervention and follow-up.

IRI score	Preinter-vention M (SD)	Follow-up M (SD)	Statistics		
			*t*	*df*	*p*
Total	12.91 (2.30)	13.01 (2.15)	0.69	13	0.50
Personal distress	3.11 (1.17)	3.01 (1.21)	0.00	13	1.00
Empathic concern	3.61 (0.87)	3.61 (0.69)	−0.84	13	0.42
Perspective taking	2.89 (0.74)	3.03 (0.42)	−0.58	13	0.57
Fantasy scale	3.30 (1.03)	3.36 (1.01)	−0.24	13	0.82

M, mean; SD, standard deviation; IRI, The Interpersonal Reactivity Index.

**TABLE 4:** Correlation between AQ-J and changes in IRI scores.

Changes in IRI scores	Differential values
Total	0.21
Personal distress	0.06
Empathic concern	−0.01
Perspective taking	0.15
Fantasy scale	0.56*

IRI, The Interpersonal Reactivity Index. *p < 0.05.

After the intervention, all participants’ supporters responded to the following statement: “Participants learned to understand the point of view of the interviewer after the intervention.”

## 4. Discussion

In the current study, we assessed the efficacy of STUH, i.e., a social skills training program using multiple humanoid robots, including an android robot. The completion rate suggests that participants in STUH were able to continue to participate in the program without losing motivation. The establishment of a highly structured learning environment may have sustained their motivation and concentration during the program and enabled them to become familiar with the perspectives of others. Most notably, participants could enhance their sociability in a way that was generalizable to real-world settings. Interestingly, no human trainers were present in the training room. On the other hand, we did not find an improvement in empathy skills, although we did observe a relationship between the autistic traits of participants and the improvement of their empathy skills.

Social skills training program using multiple humanoid robots is a social skills training program using multiple humanoid robots that is targeted at individuals with ASC. Individuals with ASC often achieve a higher degree of task engagement through interactions with robots than through interactions with human trainees (10, 15, 17, 18). In addition, peer-to-peer learning offers significant benefits with regard to concentration (48). In this study, two participants worked in pairs, which may have stimulated their competitive natures and promoted their motivation. In light of these factors, it was natural for participants to continue to participate in the program without losing motivation and to improve their skills.

In this study, two participants worked in pairs, which may have allowed participants to understand that their thoughts are different from those of others. A previous study (25) demonstrated positive results when participants worked in pairs to understand other people’s points of view, which may explain our findings. Further, this supports the notion that participants with ASC find it easier to understand that their thoughts are different from those of others when working in pairs. Although it was assumed that participants working in pairs may be more competitive than those working alone, no evidence of this was observed in both the preliminary and present studies. This suggests that participants simply enjoyed the study, which may have prevented any competitiveness. In addition, participants worked on the program from the perspective of an outsider in the first half-session, after which they simulated the experience by using robots as their avatar in the second half-session, which may have promoted their understanding of the perspectives of others and allowed them to learn and practice social skills. In fact, a previous study suggested that using multiple robots from the perspective of an outsider enables the participant to put himself in the position of each robot’ and to become familiar with the perspectives of others (16). This study (16) also revealed that the experience of using robots as avatars is also a trigger for understanding the perspectives of others.

In this study, participants were able to improve their sociability in a way that was generalizable to real-world settings. It is generally difficult for individuals with ASC to improve their skills that can be generalized to real-world settings (49). Simulating such experience by using robots as avatars in the second half-session may promote such generalization. Most notably, the appearance and motion of A-Lab android ST are similar to those of humans. Creating intelligent three-dimensional learning environments using an android robot may contribute to the development of skills that can be generalized in the real world (25, 26). Consistent with previous research (25, 26), our study showed that the intervention utilizing an android robot resulted in generalization to real-world settings to some extent. The subsequent phase of investigation would examine whether these skills transfers observable improvements in real-world situations. It is expected that the participants’ reactions will improve as the speed of the intervention delivery increase. Future studies should be conducted to further enhance the speed of the robots.

In light of the fact that the fantasy scale in the IRI is correlated with the cognitive aspect of empathy (40), this study suggested that the higher the participant’s AQ scores, the more their empathy skills improved. In general, it is difficult for individuals with stronger autistic traits to guess the position of the other person in interpersonal situations requiring empathy skills. A previous study suggested that individuals with higher AQ scores prefer android robots (27). These affinities may explain the results of this study.

The intervention associated with STUH was conducted in a space featuring no human trainers, which is beneficial with respect to maintaining social distancing in the pandemic era. In our teleoperating system, the operator can control the robot from everywhere in the world, which is beneficial in terms of recruiting human resources, especially in rural areas with few human trainers. Most notably, in light of the greater affinity of individuals with ASC with robots than with humans (13–19), the fact that no human supporters were present in the experience room is highly significant.

The study possesses some notable strengths. First, the intervention was conducted only using the robot, and all learnings to acquire the perspective of others were conducted using the robot. Second, we also simulated social situations by using the robot as an avatar. Third, we conducted these learning activities in a peer-to-peer setting. These strengths have been linked to the improvement of sociability in participants.

It should be recognized that this study has several limitations. First, the number of participants was relatively small. In addition, most participants were male. Future studies with larger samples including female participants are needed to offer more meaningful data on the potential use of this training. Second, this study was not a controlled study. We did not include a human trainer comparison group for comparison. At the time this experiment was conducted, the Japanese government had declared a state of emergency due to the spread of COVID-19, so we could not ask the participants to participate in comparison settings. Given that establishing effective SST is a pressing problem that can prevent individuals with ASC from obtaining and maintaining a competitive position, it was necessary for us to conduct pilot studies without a comparison. Since the expenses of providing care for individuals with ASC is very high (50), supporting these individuals in the task of obtaining and maintaining a competitive position is of high economic importance. To examine whether STUH can enable us to achieve this goal, future studies on long-term longitudinal designs for employment support facilities is needed.

Unlike previous studies (25, 26, 42), this study is the first to evaluate the effect of social skills training programs using multiple humanoid robots, including an android robot. Our program enabled participants to become familiar with the perspectives of others and improve their sociability in a way that was generalizable to real-world settings. Interestingly, no human trainers were present in the training room. These results contribute to making social skill training in new world. Given the promising results of this study, further studies with long-term follow-up should be conducted to draw definitive conclusions regarding the efficacy of our training system.

## Data availability statement

The raw data are available from the corresponding authors on reasonable request.

## Ethics statement

The studies involving human participants were reviewed and approved by the ethics committee of Kanazawa University. Written informed consent to participate in this study was provided by the participants’ legal guardian/next of kin. Written informed consent was obtained from the individuals and/or minors’ legal guardian for the publication of any potentially identifiable images or data included in this article.

## Author contributions

KT and HK designed the study, conducted the experiment, conducted the statistical analyses, analyzed and interpreted the data, and drafted the manuscript. TM, YY, YM, HI, and MM conceptualized the study, participated in its design, assisted with the data collection and scoring of the behavioral measures, analyzed and interpreted the data, were involved in drafting the manuscript, and revised the manuscript critically for important intellectual content. HK was involved in the final approval of the version to be published. All authors read and approved the final manuscript.
