# Supplementary material for: Social skills training using multiple humanoid robots for individuals with autism spectrum conditions
Source: Front Psychiatry. 2023 Jul 19;14:1168837. doi: 10.3389/fpsyt.2023.1168837 (PMC10394831; doi:10.3389/fpsyt.2023.1168837)
Supplement: Supplementary file 1 [file Data_Sheet_1.DOCX]

Supplementary Material 1

STUH includes four themes: emotion guessing in a one-on-one conversational situation, emotion guessing in a multiperson conversational situation, interpersonal manner, and empathic response to a person in distress. An example exercise for each theme is presented below.

| **Practice themes** | **Example of training** |
| --- | --- |
| **Emotion guessing in one-on-one conversational situation** | ***Situation***  Conversation between a teacher and a student at school.  ***Role***  A-Lab android ST: Teacher  CommU: Student  Sota: Host  ***Dialog***  **Host**: The student was called in by a teacher because he forgot many things at school.  **Student**: I’m sorry…  **Teacher**: You forget too many things. What’s wrong with you? (with angry face)  **Student**: (displays a sad face and lowers his gaze)  **Host**: How does the student feel now? Let’s imagine it based on their conversation, movements and facial expressions.  **Participant 1**: The student was looking down.  **Participant 2**: The teacher’s facial expression was different from usual.  **Participant 1**: Maybe the student was sad because he was scolded by the teacher. |
| **Emotion guessing in a multiperson conversational situation** | ***Situation***  Conversation at a part-time job  ***Role***  A-Lab android ST: Manager  CommU: Part-time worker  Sota: Host  ***Dialog***  **Host**: Someone has pushed his mistakes on a part-time worker. He has repeatedly forced the part-time worker to take the blame for his mistakes, and the part-time worker has become troubled. Finally, the manager called in the part-time worker.  **Manager**: You made a mistake again. You make too many mistakes  **Part-timer**: No, I don’t…  **Manager**: I don’t want to hear your excuse!  **Part-timer**: Eh… but it’s really not me…  **Manager**: Huh… I can’t leave anything to you like this.  **Participant 1**: Why does the manager get angry at the part-time worker when the part-time worker is forced to take the blame for the mistakes?  **Participant 2**: Does the manager think the part-time worker made a mistake?  **Participant 1**: I see, the manager does not know that the part-time worker is forced to take the blame! |
| **Interpersonal manner** | ***Situation***  Conversation between friends in a classroom.  ***Role***  A-Lab android ST: Miss Ando  CommU: Miss Sato  Sota: Host  ***Dialog***  **Host**: In the morning, Miss Ando says hello to Miss Sato in the classroom  **Miss Ando**: Good morning, Sato  **Miss Sato**: Good morning (without looking at her face)  **Ando:** (displays an annoyed face)  **Host**: Miss Ando felt somewhat uncomfortable. Why did she feel this way? And how should Miss Sato have said hello to Miss Ando? Let’s imagine.  **Participant 1**: Miss Sato greeted Miss Ando without looking at her.  **Participant 2**: Yes, I do not like to be greeted without seeing my face too.  **Participant 1**: Maybe I should look at the other person's face when I return the greeting. |
| **Empathic response to a person in distress** | ***Situation***  **Miss Ando is talking to Miss Sato about a problem**  ***Role***  A-Lab android ST: Miss Ando  CommU: Miss Sato  Sota: Host  ***Dialog***  **Miss Ando**: I was taking my dog for a walk, and when I took my eyes off it for a moment, he went somewhere. I looked everywhere for him, but I couldn't find him at all. What if I can't find him?  **Miss Sato**: ・・・  **Host**: Let's try to answer Ando's concerns sympathetically. Think about how you would feel if you were in her shoes and how she would want you to address her.  **Participant 1**: I would be sad if my pets run away. Maybe Miss Ando is sad too.  **Participant 2**: I would be sad too. But if someone encourages me, it might cheer me up.  **Participant 1**: Then, maybe I should cheer Miss Ando up, too. |
